# Identification of a *BRCA2*-Specific Modifier Locus at 6p24 Related to Breast Cancer Risk

**DOI:** 10.1371/journal.pgen.1003173

**Published:** 2013-03-27

**Authors:** Mia M. Gaudet, Karoline B. Kuchenbaecker, Joseph Vijai, Robert J. Klein, Tomas Kirchhoff, Lesley McGuffog, Daniel Barrowdale, Alison M. Dunning, Andrew Lee, Joe Dennis, Sue Healey, Ed Dicks, Penny Soucy, Olga M. Sinilnikova, Vernon S. Pankratz, Xianshu Wang, Ronald C. Eldridge, Daniel C. Tessier, Daniel Vincent, Francois Bacot, Frans B. L. Hogervorst, Susan Peock, Dominique Stoppa-Lyonnet, KConFab Investigators, Paolo Peterlongo, Rita K. Schmutzler, Katherine L. Nathanson, Marion Piedmonte, Christian F. Singer, Mads Thomassen, Ontario Cancer Genetics Network, Thomas v. O. Hansen, Susan L. Neuhausen, Ignacio Blanco, Mark H. Greene, Judith Garber, Jeffrey N. Weitzel, Irene L. Andrulis, David E. Goldgar, Emma D'Andrea, Trinidad Caldes, Heli Nevanlinna, Ana Osorio, Elizabeth J. van Rensburg, Adalgeir Arason, Gad Rennert, Ans M. W. van den Ouweland, Annemarie H. van der Hout, Carolien M. Kets, Cora M. Aalfs, Juul T. Wijnen, Margreet G. E. M. Ausems, HEBON, EMBRACE, Debra Frost, Steve Ellis, Elena Fineberg, Radka Platte, D. Gareth Evans, Chris Jacobs, Julian Adlard, Marc Tischkowitz, Mary E. Porteous, Francesca Damiola, GEMO Study Collaborators, Lisa Golmard, Laure Barjhoux, Michel Longy, Muriel Belotti, Sandra Fert Ferrer, Sylvie Mazoyer, Amanda B. Spurdle, Siranoush Manoukian, Monica Barile, Maurizio Genuardi, Norbert Arnold, Alfons Meindl, Christian Sutter, Barbara Wappenschmidt, Susan M. Domchek, Georg Pfeiler, Eitan Friedman, Uffe Birk Jensen, Mark Robson, Sohela Shah, Conxi Lazaro, Phuong L. Mai, Javier Benitez, Melissa C. Southey, Marjanka K. Schmidt, Peter A. Fasching, Julian Peto, Manjeet K. Humphreys, Qin Wang, Kyriaki Michailidou, Elinor J. Sawyer, Barbara Burwinkel, Pascal Guénel, Stig E. Bojesen, Roger L. Milne, Hermann Brenner, Magdalena Lochmann, The GENICA Network, Kristiina Aittomäki, Thilo Dörk, Sara Margolin, Arto Mannermaa, Diether Lambrechts, Jenny Chang-Claude, Paolo Radice, Graham G. Giles, Christopher A. Haiman, Robert Winqvist, Peter Devillee, Montserrat García-Closas, Nils Schoof, Maartje J. Hooning, Angela Cox, Paul D. P. Pharoah, Anna Jakubowska, Nick Orr, Anna González-Neira, Guillermo Pita, M. Rosario Alonso, Per Hall, Fergus J. Couch, Jacques Simard, David Altshuler, Douglas F. Easton, Georgia Chenevix-Trench, Antonis C. Antoniou, Kenneth Offit

**Affiliations:** 1 Epidemiology Research Program, American Cancer Society, Atlanta, Georgia, United States of America; 2 Centre for Cancer Genetic Epidemiology, Department of Public Health and Primary Care, University of Cambridge, Cambridge, United Kingdom; 3 Clinical Genetics Service, Memorial Sloan-Kettering Cancer Center, New York, New York, United States of America; 4 Program in Cancer Biology and Genetics, Memorial Sloan-Kettering Cancer Center, New York, New York, United States of America; 5 Division of Epidemiology, Department of Environmental Medicine, New York University School of Medicine, New York, New York, United States of America; 6 Centre for Cancer Genetic Epidemiology, Department of Oncology, University of Cambridge, Cambridge, United Kingdom; 7 Genetics and Population Health Division, Queensland Institute of Medical Research, Brisbane, Australia; 8 Cancer Genomics Laboratory, Centre Hospitalier Universitaire de Québec and Laval University, Québec City, Québec, Canada; 9 Unité Mixte de Génétique Constitutionnelle des Cancers Fréquents, Hospices Civils de Lyon–Centre Léon Bérard, Lyon, France; 10 INSERM U1052, CNRS UMR5286, Université Lyon 1, Centre de Recherche en Cancérologie de Lyon, Lyon, France; 11 Department of Health Sciences Research, Mayo Clinic, Rochester, Minnesota, United States of America; 12 Department of Laboratory Medicine and Pathology, Mayo Clinic, Rochester, Minnesota, United States of America; 13 Department of Epidemiology, Rollins School of Public Health, Emory University, Atlanta, Georgia, United States of America; 14 Centre d'Innovation Génome Québec et Université McGill, Montreal, Québec, Canada; 15 Family Cancer Clinic, Netherlands Cancer Institute, Amsterdam, The Netherlands; 16 Institut Curie, Department of Tumour Biology, Paris, France; 17 Institut Curie, INSERM U830, Paris, France; 18 Université Paris Descartes, Sorbonne Paris Cité, Paris, France; 19 Kathleen Cuningham Consortium for Research into Familial Breast Cancer–Peter MacCallum Cancer Center, Melbourne, Australia; 20 Unit of Molecular Bases of Genetic Risk and Genetic Testing, Department of Preventive and Predictive Medicine, Fondazione IRCCS Istituto Nazionale Tumori, Milan, Italy; 21 IFOM, Fondazione Istituto FIRC di Oncologia Molecolare, Milan, Italy; 22 University Hospital of Cologne, Cologne, Germany; 23 Abramson Cancer Center, The University of Pennsylvania School of Medicine, Philadelphia, Pennsylvania, United States of America; 24 Department of Medicine, The University of Pennsylvania School of Medicine, Philadelphia, Pennsylvania, United States of America; 25 Gynecologic Oncology Group Statistical and Data Center, Roswell Park Cancer Institute, Buffalo, New York, United States of America; 26 Department of Obstetrics and Gyncology and Comprehensive Cancer Center, Medical University of Vienna, Vienna, Austria; 27 Department of Clinical Genetics, Odense University Hospital, Odense, Denmark; 28 Samuel Lunenfeld Research Institute, Mount Sinai Hospital, Toronto, Ontario, Canada; 29 Center for Genomic Medicine, Rigshospitalet, Copenhagen University Hospital, Copenhagen, Denmark; 30 Department of Population Sciences, Beckman Research Institute of City of Hope, Duarte, California, United States of America; 31 Genetic Counseling Unit, Hereditary Cancer Program, IDIBELL–Catalan Institute of Oncology, Barcelona, Spain; 32 Clinical Genetics Branch, Division of Cancer Epidemiology and Genetics, National Cancer Institute, National Institutes of Health, Rockville, Maryland, United States of America; 33 Department of Medical Oncology, Dana-Farber/Partners CancerCare, Boston, Massachusetts, United States of America; 34 Clinical Cancer Genetics (for the City of Hope Clinical Cancer Genetics Community Research Network), City of Hope, Duarte, California, United States of America; 35 Samuel Lunenfeld Research Institute, Mount Sinai Hospital, Toronto, Ontario, Canada; 36 Departments of Molecular Genetics and Laboratory Medicine and Pathobiology, University of Toronto, Ontario, Canada; 37 Department of Dermatology, University of Utah School of Medicine, Salt Lake City, Utah, United States of America; 38 Department of Surgery, Oncology, and Gastroenterology, University of Padua, Padua, Italy; 39 Immunology and Molecular Oncology Unit, Istituto Oncologico Veneto IOV-IRCCS, Padua, Italy; 40 Molecular Oncology Laboratory, Hospital Clinico San Carlos, IdISSC, Madrid, Spain; 41 Department of Obstetrics and Gynecology, University of Helsinki and Helsinki University Central Hospital, Helsinki, Finland; 42 Human Genetics Group, Spanish National Cancer Centre (CNIO), Madrid, Spain; 43 Biomedical Network on Rare Diseases (CIBERER), Madrid, Spain; 44 Department of Genetics, University of Pretoria, Pretoria, South Africa; 45 Department of Pathology, Landspitali University Hospital, Reykjavik, Iceland; 46 BMC, Faculty of Medicine, University of Iceland, Reykjavik, Iceland; 47 Clalit National Israeli Cancer Control Center and Department of Community Medicine and Epidemiology, Carmel Medical Center and B. Rappaport Faculty of Medicine, Haifa, Israel; 48 Department of Clinical Genetics, Family Cancer Clinic, Erasmus University Medical Center, Rotterdam, The Netherlands; 49 Department of Genetics, University Medical Center, Groningen University, Groningen, The Netherlands; 50 Department of Human Genetics, Radboud University Nijmegen Medical Centre, Nijmegen, The Netherlands; 51 Department of Clinical Genetics, Academic Medical Center, Amsterdam, The Netherlands; 52 Department of Human Genetics and Department of Clinical Genetics, Leiden University Medical Center, Leiden, The Netherlands; 53 Department of Medical Genetics, University Medical Center Utrecht, Utrecht, The Netherlands; 54 Department of Epidemiology, Netherlands Cancer Institute, Amsterdam, The Netherlands; 55 Clinical Genetics, Guy's and St. Thomas' NHS Foundation Trust, London, United Kingdom; 56 Yorkshire Regional Genetics Service, Leeds, United Kingdom; 57 Department of Medical Genetics, University of Cambridge, Cambridge, United Kingdom; 58 South East of Scotland Regional Genetics Service, Western General Hospital, Edinburgh, United Kingdom; 59 National Cancer Genetics Network, UNICANCER Genetic Group, France; 60 Cancer Genetics Unit, INSERM U916, Institut Bergonié, Université de Bordeaux, Bordeaux, France; 61 Laboratoire de Génétique Chromosomique, Hôtel Dieu Centre Hospitalier, Chambéry, France; 62 Unit of Medical Genetics, Department of Preventive and Predictive Medicine, Fondazione IRCCS Istituto Nazionale Tumori (INT), Milan, Italy; 63 Division of Cancer Prevention and Genetics, Istituto Europeo di Oncologia, Milan, Italy; 64 Fiorgen Foundation for Pharmacogenomics and Unit of Medical Genetics, Department of Clinical Physiopathology, University of Florence, Florence, Italy; 65 University Hospital of Schleswig-Holstein, University Kiel, Kiel, Germany; 66 Department of Gynaecology and Obstetrics, Division of Tumor Genetics, Klinikum rechts der Isar, Technical University, Munich, Germany; 67 University of Heidelberg, Heidelberg, Germany; 68 Sheba Medical Center, Tel Aviv, Israel; 69 Department of Clinical Genetics, Aarhus University Hospital, Aarhus, Denmark; 70 Molecular Diagnostic Unit, Hereditary Cancer Program, IDIBELL–Catalan Institute of Oncology, Barcelona, Spain; 71 Centre for Molecular, Environmental, Genetic, and Analytic Epidemiology, The University of Melbourne, Melbourne, Australia; 72 Netherlands Cancer Institute, Antoni van Leeuwenhoek Hospital, Amsterdam, The Netherlands; 73 University Breast Center Franconia, Department of Gynecology and Obstetrics, University Hospital Erlangen, Erlangen, Germany; 74 Department of Medicine, Division of Hematology and Oncology, David Geffen School of Medicine, University of California Los Angeles, Los Angeles, California, United States of America; 75 London School of Hygiene and Tropical Medicine, London, United Kingdom; 76 Division of Cancer Studies, NIHR Comprehensive Biomedical Research Centre, Guy's & St. Thomas' NHS Foundation Trust in partnership with King's College London, London, United Kingdom; 77 Department of Obstetrics and Gynecology, University of Heidelberg, Heidelberg, Germany; 78 Molecular Epidemiology Group, German Cancer Research Center (DKFZ), Heidelberg, Germany; 79 Inserm (National Institute of Health and Medical Research), CESP (Center for Research in Epidemiology and Population Health), U1018, Environmental Epidemiology of Cancer, Villejuif, France; 80 University of Paris-Sud, UMR-S 1018, Villejuif, France; 81 Copenhagen General Population Study and Department of Clinical Biochemistry, Herlev Hospital, Copenhagen University Hospital, University of Copenhagen, Copenhagen, Denmark; 82 Division of Clinical Epidemiology and Aging Research, German Cancer Research Center, Heidelberg, Germany; 83 Dr. Margarete Fischer-Bosch-Institute of Clinical Pharmacology, Stuttgart, Germany; 84 University of Tübingen, Tübingen, Germany; 85 Molecular Genetics of Breast Cancer, Deutsches Krebsforschungszentrum (DKFZ), Heidelberg, Germany; 86 Institute for Prevention and Occupational Medicine of the German Social Accident Insurance (IPA), Bochum, Germany; 87 Institute and Outpatient Clinic of Occupational Medicine, Saarland University Medical Center and Saarland University Faculty of Medicine, Homburg, Germany; 88 Institute of Pathology, Medical Faculty of the University of Bonn, Bonn, Germany; 89 Department of Internal Medicine, Evangelische Kliniken Bonn gGmbH, Johanniter Krankenhaus, Bonn, Germany; 90 Department of Clinical Genetics, Helsinki University Central Hospital, Helsinki, Finland; 91 Department of Obstetrics and Gynecology, Hannover Medical School, Hannover, Germany; 92 Department of Oncology and Pathology, Karolinska Institute, Stockholm, Sweden; 93 School of Medicine, Institute of Clinical Medicine, Pathology, and Forensic Medicine, Biocenter Kuopio, Cancer Center of Eastern Finland, University of Eastern Finland, Kuopio, Finland; 94 Imaging Center, Department of Clinical Pathology, Kuopio University Hospital, Kuopio, Finland; 95 Vesalius Research Center, VIB, Leuven, Belgium; 96 Laboratory for Translational Genetics, Department of Oncology, University of Leuven, Belgium; 97 Division of Cancer Epidemiology, German Cancer Research Center (DKFZ), Heidelberg, Germany; 98 Cancer Epidemiology Centre, The Cancer Council Victoria, Melbourne, Australia; 99 Centre for Molecular, Environmental, Genetic, and Analytic Epidemiology, The University of Melbourne, Melbourne, Australia; 100 Department of Preventive Medicine, Keck School of Medicine, University of Southern California, Los Angeles, California, United States of America; 101 Laboratory of Cancer Genetics and Tumor Biology, Department of Clinical Genetics and Biocenter Oulu, University of Oulu, Oulu University Hospital, Oulu, Finland; 102 Department of Human Genetics and Department of Pathology, Leiden University Medical Center, Leiden, The Netherlands; 103 Division of Genetics and Epidemiology and Division of Breast Cancer Research, The Institute of Cancer Research, Sutton, United Kingdom; 104 Division of Breast Cancer Research, Breakthrough Breast Cancer Research Centre, Institute of Cancer Research, London, United Kingdom; 105 Medical Epidemiology and Biostatistics, Karolinska Institutet, Stockholm, Sweden; 106 Department of Medical Oncology, Erasmus University Medical Center, Rotterdam, The Netherlands; 107 CRUK/YCR Sheffield Cancer Research Centre, Department of Oncology, University of Sheffield, Sheffield, United Kingdom; 108 Department of Genetics and Pathology, Pomeranian Medical University, Szczecin, Poland; 109 Human Genotyping–CEGEN Unit, Human Cancer Genetics Program, Spanish National Cancer Research Centre [CNIO], Madrid, Spain; 110 Department of Molecular Biology and Medicine, Massachusetts General Hospital, Boston, Massachusetts, United States of America; 111 Program in Medical and Population Genetics, Broad Institute of Harvard and MIT, Cambridge, Massachusetts, United States of America; 112 Departments of Genetics and Medicine, Harvard Medical School, Boston, Massachusetts, United States of America; National Cancer Institute, United States of America

## Abstract

Common genetic variants contribute to the observed variation in breast cancer risk for *BRCA2* mutation carriers; those known to date have all been found through population-based genome-wide association studies (GWAS). To comprehensively identify breast cancer risk modifying loci for *BRCA2* mutation carriers, we conducted a deep replication of an ongoing GWAS discovery study. Using the ranked P-values of the breast cancer associations with the imputed genotype of 1.4 M SNPs, 19,029 SNPs were selected and designed for inclusion on a custom Illumina array that included a total of 211,155 SNPs as part of a multi-consortial project. DNA samples from 3,881 breast cancer affected and 4,330 unaffected *BRCA2* mutation carriers from 47 studies belonging to the Consortium of Investigators of Modifiers of *BRCA1/2* were genotyped and available for analysis. We replicated previously reported breast cancer susceptibility alleles in these *BRCA2* mutation carriers and for several regions (including *FGFR2*, *MAP3K1*, *CDKN2A/B*, and *PTHLH*) identified SNPs that have stronger evidence of association than those previously published. We also identified a novel susceptibility allele at 6p24 that was inversely associated with risk in *BRCA2* mutation carriers (rs9348512; per allele HR = 0.85, 95% CI 0.80–0.90, P = 3.9×10^−8^). This SNP was not associated with breast cancer risk either in the general population or in *BRCA1* mutation carriers. The locus lies within a region containing *TFAP2A*, which encodes a transcriptional activation protein that interacts with several tumor suppressor genes. This report identifies the first breast cancer risk locus specific to a *BRCA2* mutation background. This comprehensive update of novel and previously reported breast cancer susceptibility loci contributes to the establishment of a panel of SNPs that modify breast cancer risk in *BRCA2* mutation carriers. This panel may have clinical utility for women with *BRCA2* mutations weighing options for medical prevention of breast cancer.

## Author Summary

Women who carry *BRCA2* mutations have an increased risk of breast cancer that varies widely. To identify common genetic variants that modify the breast cancer risk associated with *BRCA2* mutations, we have built upon our previous work in which we examined genetic variants across the genome in relation to breast cancer risk among *BRCA2* mutation carriers. Using a custom genotyping platform with 211,155 genetic variants known as single nucleotide polymorphisms (SNPs), we genotyped 3,881 women who had breast cancer and 4,330 women without breast cancer, which represents the largest possible, international collection of *BRCA2* mutation carriers. We identified that a SNP located at 6p24 in the genome was associated with lower risk of breast cancer. Importantly, this SNP was not associated with breast cancer in *BRCA1* mutation carriers or in a general population of women, indicating that the breast cancer association with this SNP might be specific to *BRCA2* mutation carriers. Combining this *BRCA2*-specific SNP with 13 other breast cancer risk SNPs also known to modify risk in *BRCA2* mutation carriers, we were able to derive a risk prediction model that could be useful in helping women with *BRCA2* mutations weigh their risk-reduction strategy options.

## Introduction

The lifetime risk of breast cancer associated with carrying a *BRCA2* mutation varies from 40 to 84% [1]. To determine whether common genetic variants modify breast cancer risk for *BRCA2* mutation carriers, we previously conducted a GWAS of *BRCA2* mutation carriers from the Consortium of Investigators of Modifiers of *BRCA1/2* (CIMBA) [2]. Using the Affymetrix 6.0 platform, the discovery stage results were based on 899 young (<40 years) affected and 804 unaffected carriers of European ancestry. In a rapid replication stage wherein 85 discovery stage SNPs with the smallest P-values were genotyped in 2,486 additional *BRCA2* mutation carriers, only published loci associated with breast cancer risk in the general population, including *FGFR2* (10q26; rs2981575; P = 1.2×10^−8^), were associated with breast cancer risk at the genome-wide significance level among *BRCA2* mutation carriers. Two other loci, in *ZNF365* (rs16917302) on 10q21 and a locus on 20q13 (rs311499), were also associated with breast cancer risk in *BRCA2* mutation carriers with P-values<10^−4^ (P = 3.8×10^−5^ and 6.6×10^−5^, respectively). A nearby SNP in *ZNF365* was also associated with breast cancer risk in a study of unselected cases [3] and in a study of mammographic density [4]. Additional follow-up replicated the findings for rs16917302, but not rs311499 [5] in a larger set of *BRCA2* mutation carriers. To seek additional breast cancer risk modifying loci for *BRCA2* mutation carriers, we conducted an extended replication of the GWAS discovery results in a larger set of *BRCA2* mutation carriers in CIMBA, which represents the largest, international collection of *BRCA2* mutation carriers.

## Materials and Methods

### Ethics statement

Each of the host institutions (Table S1) recruited under ethically-approved protocols. Written informed consent was obtained from all subjects.

### Study subjects

The majority of *BRCA2* mutation carriers were recruited through cancer genetics clinics and some came from population or community-based studies. Studies contributing DNA samples to these research efforts were members of the Consortium of Investigators of Modifiers of *BRCA1/2* (CIMBA) with the exception of one study (NICCC). Eligible subjects were women of European descent who carried a pathogenic *BRCA2* mutation, had complete phenotype information, and were at least 18 years of age. Harmonized phenotypic data included year of birth, age at cancer diagnosis, age at bilateral prophylactic mastectomy and oophorectomy, age at interview or last follow-up, *BRCA2* mutation description, self-reported ethnicity, and breast cancer estrogen receptor status.

#### GWAS discovery stage samples

Details of these samples have been described previously [2]. Data from 899 young (<40 years) affected and 804 older (>40 years) unaffected carriers of European ancestry from 14 countries were used to select SNPs for inclusion on the iCOGS array.

#### Samples genotyped in the extended replication set

Forty-seven studies from 24 different countries (including two East-Asian countries) provided DNA from a total of 10,048 *BRCA2* mutations carriers. All eligible samples were genotyped using COGs, including those from the discovery stage.

### Genotyping and quality control

#### *BRCA2* SNP selection for inclusion on iCOGS

The Collaborative Oncological Gene-Environment Study (COGS) consortium developed a custom genotyping array (referred to as the iCOGS array) to provide efficient genotyping of common and rare genetic variants to identify novel loci that are associated with risk of breast, ovarian, and prostate cancers as well as to fine-map known cancer susceptibility loci. SNPs were selected for inclusion on iCOGS separately by each participating consortium: Breast Cancer Association Consortium (BCAC) [6], Ovarian Cancer Association Consortium (OCAC) [7], Prostate Cancer Association Group to Investigate Cancer Associated Alterations in the Genome (PRACTICAL) [8], and CIMBA. SNP lists from a *BRCA1* GWAS and SNPs in candidate regions were used together with the *BRCA2* GWAS lists to generate a ranked CIMBA SNP list that included SNPs with the following nominal proportions: 55.5% from the *BRCA1* GWAS, 41.6% from the *BRCA2* GWAS and fine mapping, 2.9% for CIMBA candidate SNPs. Each consortium was given a share of the array: nominally 25% of the SNPs each for BCAC, PRACTICAL and OCAC; 17.5% for CIMBA; and 7.5% for SNPs from commonly researched pathways (e.g., inflammation). For the CIMBA *BRCA2* GWAS, we used the iCOGS array as the platform to genotype the extended replication set of the discovery GWAS stage [2]. SNPs were selected on the basis of the strength of their associations with breast cancer risk in the discovery stage [2], using imputed genotype data for 1.4 M SNPs identified through CEU+TSI samples on HapMap3, release 2. A ranked list of SNPs was based on the 1-df trend test statistic, after excluding highly correlated SNPs (r^2^>0.4). The final list included the 39,015 SNPs with the smallest p-values. An additional set of SNPs were selected for fine mapping of the regions surrounding the SNPs found to be associated with breast cancer in the discovery GWAS stage: rs16917302 on 10q21 and rs311499 on 20q13, including SNPs with a MAF >0.05 located 500 kb in both directions of the SNP, based on HapMap 2 data. The final combined list of SNPs for the iCOGS array comprised 220,123 SNPs. Of these, 211,155 were successfully manufactured onto the array. The present analyses are based on the 19,029 SNPs selected on the basis of *BRCA2* GWAS and fine mapping that were included on the iCOGS array.

#### Genotyping

The genotyping was performed on DNA samples from 10,048 *BRCA2* mutation carriers at the McGill University and Génome Québec Innovation Centre (Montreal, Canada). As a quality control measure, each plate included DNA samples from six individuals who were members of two CEPH trios. Some plates also contained three duplicate pairs of quality control samples. Genotypes were called using GenCall [9]. Initial calling was based on a cluster file generated using 270 samples from Hapmap2. To generate the final calls, we first selected a subset of 3,018 individuals, including samples from each of the genotyping centers in the iCOGS project, each of the participating consortia, and each major ethnicity. Only plates with a consistent high call rate in the initial calling were used. We also included 380 samples of European, African, and Asian ethnicity genotyped as part of the Hapmap and 1000 Genomes project, and 160 samples that were known positive controls for rare variants on the array. This subset was used to generate a cluster file that was then applied to call the genotypes for the remaining samples.

#### Quality control of SNPs

Of the 211,155 SNPs on the iCOGS array, we excluded SNPs for the following reasons (Table S2): on the Y-chromosome, call rate <95%, deviations from Hardy-Weinberg equilibrium (P<10^−7^) using a stratified 1-d.f. test [10], and monomorphic. SNPs that gave discrepant genotypes among known duplicates were also excluded. After quality control filtering, 200,908 SNPs were available for analysis (Table S2); 18,086 of which were selected on the basis of the discovery *BRCA2* GWAS [2]. Cluster plots of all reported SNPs were inspected manually for quality (Figure S1).

#### Description of imputation

Genotypes for SNPs identified through the 1000 Genomes Phase I data (released Jan 2012) [11] were imputed using all SNPs on the iCOGS chip in a region of 500 kb around the novel modifier locus at 6p24. The boundaries were determined according to the linkage disequilibrium (LD) structure in the region based on HapMap data. The imputation was carried out using IMPUTE 2.2 [12]. SNPs with imputation information/accuracy r^2^<0.30 were excluded in the analyses.

#### Quality control of DNA samples

Of 10,048 genotyped samples (Table S2), 742 were excluded because they did not meet the phenotypic eligibility criteria or had self-reported non-CEU ethnicity. Samples were then excluded for the following reasons: not female (XXY, XY), call rate <95%, low or high heterozygosity (P<10^−6^), discordant genotypes from previous CIMBA genotyping efforts, or discordant duplicate samples. For duplicates with concordant phenotypic data, or in cases of cryptic monozygotic twins, only one of the samples was included. Cryptic duplicates for which phenotypic data indicated different individuals were all excluded. Samples of non-European ancestry were identified using multi-dimensional scaling, after combining the *BRCA2* mutation carrier samples with the HapMap2 CEU, CHB, JPT and YRI samples using a set of 37,120 uncorrelated SNPs from the iCOGS array. Samples with >19% non-European ancestry were excluded (Figure S2). A total of 4,330 affected and 3,881 unaffected *BRCA2* mutation carrier women of European ancestry from 42 studies remained in the analysis (Table S1), including 3,234 breast cancer cases and 3,490 unaffected carriers that were not in the discovery set.

#### *BRCA1* and BCAC samples

Details of the sample collection, genotyping and quality control process for the *BRCA1* and BCAC samples, are reported elsewhere [13], [14].

### Statistical methods

The associations between genotype and breast cancer risk were analyzed within a retrospective cohort framework with time to breast cancer diagnosis as the outcome [15]. Each *BRCA2* carrier was followed until the first event: breast or ovarian cancer diagnosis, bilateral prophylactic mastectomy, or age at last observation. Only those with a breast cancer diagnosis were considered as cases in the analysis. The majority of mutation carriers were recruited through genetic counseling centers where genetic testing is targeted at women diagnosed with breast or ovarian cancer and in particular to those diagnosed with breast cancer at a young age. Therefore, these women are more likely to be sampled compared to unaffected mutation carriers or carriers diagnosed with the disease at older ages. As a consequence, sampling was not random with respect to disease phenotype and standard methods of survival analysis (such as Cox regression) may lead to biased estimates of the associations [16]. We therefore conducted the analysis by modelling the retrospective likelihood of the observed genotypes conditional on the disease phenotypes. This has been shown to provide unbiased estimates of the associations [15]. The implementation of the retrospective likelihoods has been described in detail elsewhere [15], [17]. The associations between genotype and breast cancer risk were assessed using the 1degree of freedom score test statistic based on the retrospective likelihood [15]. In order to account for non-independence between relatives, an adjusted version of the score test was used in which the variance of the score was derived taking into account the correlation between the genotypes [18]. P-values were not adjusted using genomic control because there was little evidence of inflation. Inflation was assessed using the genomic inflation factor, λ. Since this estimate is dependent on sample size, we also calculated λ adjusted to 1000 affected and 1000 unaffected samples. Per-allele and genotype-specific hazard-ratios (HR) and 95% confidence intervals (CI) were estimated by maximizing the retrospective likelihood. Calendar-year and cohort-specific breast cancer incidences for *BRCA2* were used [1]. All analyses were stratified by country of residence. The USA and Canada strata were further subdivided by self-reported Ashkenazi Jewish ancestry. The assumption of proportional hazards was assessed by fitting a model that included a genotype-by-age interaction term. Between-country heterogeneity was assessed by comparing the results of the main analysis to a model with country-specific log-HRs. A possible survival bias due to inclusion of prevalent cases was evaluated by re-fitting the model after excluding affected carriers that were diagnosed ≥5 years prior to study recruitment. The associations between genotypes and tumor subtypes were evaluated using an extension of the retrospective likelihood approach that models the association with two or more subtypes simultaneously [19]. To investigate whether any of the significant SNPs were associated with ovarian cancer risk for *BRCA2* mutation carriers and whether the inclusion of ovarian cancer patients as unaffected subjects biased our results, we also analyzed the data within a competing risks framework and estimated HR simultaneously for breast and ovarian cancer using the methods described elsewhere [15]. Analyses were carried out in R using the GenABEL libraries [20] and custom-written software. The retrospective likelihood was modeled in the pedigree-analysis software MENDEL [21], as described in detail elsewhere [15].

#### TCGA analysis

Affymetrix SNP 6.0 genotype calls for normal (non-tumor) breast DNA were downloaded for all available individuals from The Cancer Genome Atlas in September 2011. Analyses were limited to the 401 individuals of European ancestry based on principal component analysis. Expression levels in breast tumor tissue were adjusted for the top two principal components, age, gender (there are some male breast cancer cases in TCGA), and average copy number across the gene in the tumor. Linear regression was then used to test for association between the SNP and the adjusted gene expression level for all genes within one megabase.

#### Gene set enrichment analysis

To investigate enrichment of genes associated with breast cancer risk, the gene-set enrichment approach was implemented using Versatile Gene-based Association Study [22] based on the ranked P-values from retrospective likelihood analysis. Association List Go Annotator was also used to prioritize gene pathways using functional annotation from gene ontology (GO) [23] to increase the power to detect association to a pathway, as opposed to individual genes in the pathway. Both analyses were corrected for LD between SNPs, variable gene size, and interdependence of GO categories, where applicable, based on imputation. 100,000 Monte Carlo simulations were performed in VEGAS and 5000 replicate gene lists using random sampling of SNPs and 5000 replicate studies (sampling with replacement) were performed to estimate P-values.

#### Predicted absolute breast cancer risks by combined SNP profile

We estimated the absolute risks of developing breast cancer based on the joint distribution of SNPs associated with breast cancer for *BRCA2* mutation carriers. The methods have been described elsewhere [24]. To construct the SNP profiles, we considered the single SNP from each region with the strongest evidence of association in the present dataset. We included all loci that had previously been found to be associated with breast cancer risk through GWAS in the general population and demonstrated associations with breast cancer risk for *BRCA2* mutation carriers, and loci that had GWAS level of significance in the current study. We assumed that all loci in the profile were independent (i.e. they interact multiplicatively on *BRCA2* breast cancer risk). Genotype frequencies were obtained under the assumption of Hardy-Weinberg Equilibrium. For each SNP, the effect of each allele was assumed to be consistent with a multiplicative model (log-additive). We assumed that the average, age-specific breast cancer incidences, over all associated loci, agreed with published breast cancer risk estimates for *BRCA2* mutation carriers [1].

## Results

The genomic inflation factor (λ) based on the 18,086 *BRCA2* GWAS SNPs in the 6,724 *BRCA2* mutation carriers who were not used in the SNP discovery set was 1.034 (λ adjusted to 1000 affected and 1000 unaffected: 1.010, Figure S3). Multiple variants were associated with breast cancer risk in the combined discovery and replication datasets (Figure S4). SNPs in three independent regions had P-values<5×10^−8^; one was a region not previously associated with breast cancer.

The most significant associations were observed for known breast cancer susceptibility regions, rs2420946 (per allele P = 2×10^−14^) in *FGFR2* and rs3803662 (P = 5.4×10^−11^) near *TOX3* (Table 1). Breast cancer risk associations with other SNPs reported previously for *BRCA2* mutation carriers are summarized in Table 1. In this larger set of *BRCA2* mutation carriers, we also identified novel SNPs in the 12p11 (*PTHLH*), 5q11 (*MAP3K1)*, and 9p21 (*CDKN2A/B*) regions with smaller P-values for association than those of previously reported SNPs. These novel SNPs were not correlated with the previously reported SNPs (r^2^<0.14). For one of the novel SNPs identified in the discovery GWAS [2], Z*NF365* rs16917302, there was weak evidence of association with breast cancer risk (P = 0.01); however, an uncorrelated SNP, rs17221319 (r^2^<0.01), 54 kb upstream of rs16917302 had stronger evidence of association (P = 6×10^−3^).

**Table 1:** Per allele hazard ratios (HR) and 95% confidence intervals (CI) of previously published breast cancer loci among *BRCA2* mutation carriers from previous reports and from the iCOGS array, ordered by statistical significance of the region.

Chr (Nearby Genes)	Report Status^1^	SNP	r^2^	MinorAllele	Previously Reported Results					iCOGS Results				
					Ref	Affected N	Unaffected N	Per Allele HR (95%CI)	p-value^2^	Affected N	Unaffected N	Unaffected MAF	Per Allele HR (95%CI)	p-value^2^
10q26 (*FGFR2*)	reported	rs2981575		G	[2]	2,155	2,016	1.28 (1.18, 1.39)	1×10^−8^	4,326	3,874	0.40		
	novel	rs2420946	0.96	A						4,328	3,877	0.39	1.27 (1.19, 1.34)	2×10^−14^
16q12 (*TOX3*)	reported	rs3803662		A	[2]	2,162	2,026	1.20 (1.10, 1.31)	5×10^−5^	4,330	3,880	0.27	1.24 (1.16, 1.32)	5×10^−11^
12p11 (*PTHLH*)	reported	rs10771399		G	[34]	3,798	3,314	0.93 (0.84, 1.04)	0.20	4,330	3,880	0.11	0.89 (0.81, 0.98)	0.02
	novel	rs27633	0.05	C						4,252	3,841	0.39	1.14 (1.07, 1.21)	4×10^−5^
5q11 (*MAP3K1)*	reported	rs889312		C	[24]	2,840	2,282	1.10 (1.01, 1.19)	0.02	4,330	3,881	0.29	1.04 (0.98, 1.11)	0.20
	novel	rs16886113	0.14	C						4,330	3,881	0.06	1.24 (1.11, 1.38)	1×10^−4^
9p21 (*CDKN2A/B*)	reported	rs1011970		A	[34]	3,807	3,316	1.09 (1.00, 1.18)	0.05	4,330	3,881	0.17	1.03 (0.95, 1.11)	0.51
	novel	rs10965163	0.00	A						4,329	3,880	0.10	0.84 (0.77, 0.93)	8×10^−4^
11p15 (*LSP1)*	reported	rs3817198		G	[24]	3,266	2,636	1.14 (1.06, 1.23)	8×10^−4^	4,316	3,870	0.33	1.11 (1.04, 1.18)	9×10^−4^
8q24	reported	rs13281615		G	[24]	3,338	2,723	1.06 (0.98, 1.13)	0.13	4,248	3,810	0.43	1.03 (0.97, 1.09)	0.31
	novel	rs4733664	0.00	A						4,329	3,879	0.41	1.10 (1.04, 1.17)	2×10^−3^
20q13	reported	rs311498^3^		A^4^	[5]	3,808	3,318	0.95 (0.84, 1.07)	0.36	4,330	3,880	0.07	0.95 (0.84, 1.06)	0.31
	novel	rs13039229	0.00	C						4,326	3,877	0.21	0.90 (0.84, 0.97)	5×10^−3^
6q25 (*ESR1*)	reported	rs9397435		G	[35]	3,809	3,316	1.14 (1.01, 1.27)	0.03	4,330	3,881	0.08	1.12 (1.00, 1.25)	0.03
	novel	rs2253407	0.01	A						4,330	3,881	0.47	0.92 (0.86, 0.98)	5×10^−3^
10q21 (*ZNF365*)	reported	rs16917302		C	[5]	3,807	3,315	0.83 (0.75, 0.93)	7×10^−4^	4,330	3,881	0.11	0.88 (0.80, 0.98)	0.01
	novel	rs17221319	0.00	A						4,330	3,881	0.46	1.09 (1.02, 1.15)	6×10^−3^
3p24 (*SLC4A7, NEK10*)	reported	rs4973768		A	[24]	3,370	2,783	1.10 (1.03, 1.18)	6×10^−3^	4,322	3,875	0.49	1.09 (1.02, 1.15)	7×10^−3^
12q24	reported	rs1292011^4^		G	[34]	2,530	2,342	0.94 (0.87, 1.01)	0.10	4,313	3,875	0.42	0.92 (0.87, 0.98)	0.01
5p12	reported	rs10941679^4^		G	[24]	3,263	2,591	1.09 (1.01, 1.19)	0.03	4,320	3,875	0.24	1.07 (1.01, 1.15)	0.04
11q13	reported	rs614367		A	[34]	3,789	3,307	1.03 (0.95, 1.13)	0.46	4,330	3,880	0.14	1.08 (1.00, 1.17)	0.04
1p11 (*NOTCH2*)	reported	rs11249433		G	[35]	3,423	2,827	1.09 (1.02, 1.17)	0.02	4,328	3,881	0.40	1.05 (0.99, 1.12)	0.10
17q23 (*STXBP4, COX11*)	reported	rs6504950		A	[24]	3,401	2,813	1.03 (0.95, 1.11)	0.47	4,329	3,881	0.26	1.04 (0.97, 1.11)	0.23
19p13 (*MERIT40*)	reported	rs8170		A	[5]	3,665	3,086	0.98 (0.90, 1.07)	0.66	4,327	3,876	0.19	0.98 (0.91, 1.06)	0.62
2q35	reported	rs13387042^4^		G	[24]	3,300	2,646	1.05 (0.98, 1.13)	0.14	4,326	3,880	0.48	0.99 (0.93, 1.05)	0.66
9q31	reported	rs865686		C	[34]	3,799	3,312	0.95 (0.89, 1.01)	0.10	4,330	3,880	0.36	0.99 (0.93, 1.05)	0.77
10q22 (*ZMIZ1*)	reported	rs704010		A	[34]	3,761	3,279	1.01 (0.95, 1.08)	0.73	4,328	3,878	0.38	1.01 (0.95, 1.07)	0.91

1 Reporting status of the SNP is either previously reported or novel to this report.

2 p-value was calculated based on the 1-degree of freedom score test statistic.

3 rs311499 could not be designed onto the iCOGS array. A surrogate (r^2^ = 1.0), rs311498, was included, however, and reported here.

4 Stronger associations were originally reported for the SNP, assuming a dominant or recessive model of the ‘risk allele’.

**Figure 1:**
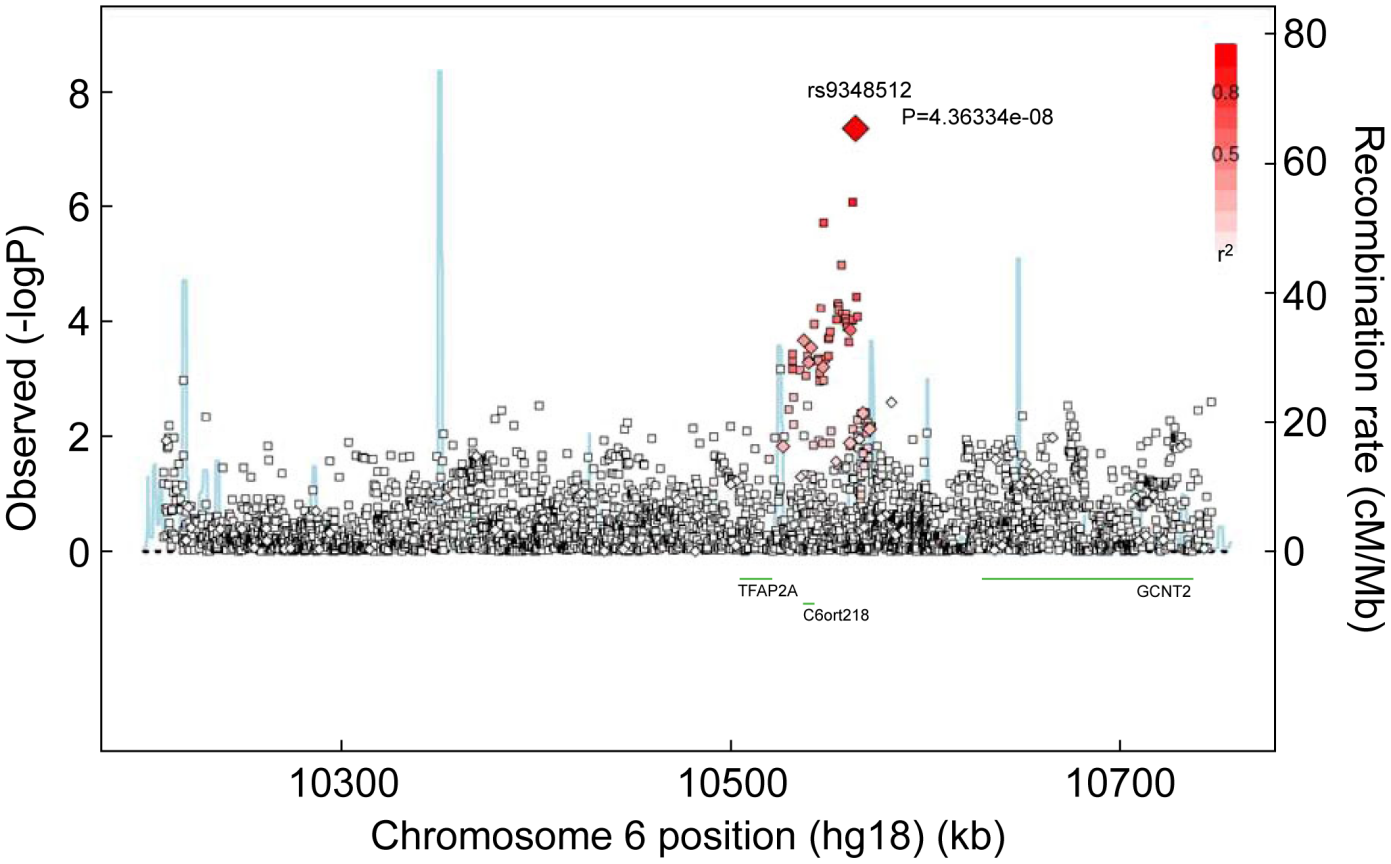
Associations between SNPs in the region surrounding rs9348512 on chromosome 6 and breast cancer risk. Results based on imputed and observed genotypes. The blue spikes indicate the recombination rate at each position. Genotyped SNPs are represented by diamonds and imputed SNPs are represented by squares. Color saturation indicates the degree of correlation with the SNP rs9348512.

One SNP, rs9348512 at 6p24 not known to be associated with breast cancer, had a combined P-value of association of 3.9×10^−8^ amongst all *BRCA2* samples (Table 2), with strong evidence of replication in the set of *BRCA2* samples that were not used in the discovery stage (P = 5.2×10^−5^). The minor allele of rs9348512 (MAF = 0.35) was associated with a 15% decreased risk of breast cancer among *BRCA2* mutation carriers (per allele HR = 0.85, 95% CI 0.80–0.90) with no evidence of between-country heterogeneity (P = 0.78, Figure S5). None of the genotyped (n = 68) or imputed (n = 3,507) SNPs in that region showed a stronger association with risk (Figure 1; Table S3), but there were 40 SNPs with P<10^−4^ (pairwise r^2^>0.38 with rs9348512, with the exception of rs11526201 for which r^2^ = 0.01, Table S3). The association with rs9348512 did not differ by 6174delT mutation status (P for difference = 0.33), age (P = 0.39), or estrogen receptor (ER) status of the breast tumor (P = 0.41). Exclusion of prevalent breast cancer cases (n = 1,752) produced results (HR = 0.83, 95% CI 0.77–0.89, P = 3.40×10^−7^) consistent with those for all cases.

**Table 2:** Breast cancer hazard ratios (HR) and 95% confidence intervals (CI) of novel breast cancer loci with P-values of association <10^−5^ among *BRCA2* mutation carriers.

		Discovery Stage				Stage 2				Combined				
SNP rs No. Chr. (Nearby Genes)	Genotype	Affected No. (%)	Unaffected No. (%)	HR (95% CI)	p-value^1^	Affected No. (%)	Unaffected No. (%)	HR (95% CI)	p-value^1^	Affected No. (%)	Unaffected No. (%)	Unaffected MAF	HR (95% CI)	p-value^1^
rs9348512 Chr6 (*TFAP2A, C6orf218)*	CC	390 (46.4)	248 (38.3)	1.00			1392 (43.0)				1640 (42.3)	0.35	1.00	
	CA	368 (43.8)	299 (46.2)	0.81 (0.67–0.96)		1515 (43.4)	1432 (44.3)	0.92 (0.83–1.01)		1883 (43.5)	1731 (44.6)		0.89 (0.82–0.97)	
	AA	82 (9.8)	100 (15.5)	0.55 (0.42–0.74)		368 (10.5)	410 (12.7)	0.72 (0.62–0.84)		450 (10.4)	510 (12.1)		0.68 (0.59–0.78)	
	per allele			0.76 (0.67–0.87)	2.6×10^−5^			0.87 (0.81–0.93)	5.2×10^−5^				0.85 (0.80–0.90)	3.9×10^−8^
rs619373 ChrX (*FGF13*)	GG	693 (75.8)	568 (87.8)	1.00		2882 (82.7)	2784 (86.1)	1.00		3575 (82.6)	3352 (86.4)	0.07	1.00	
	GA	143 (15.7)	78 (12.1)	1.43 (1.13–1.80)		583 (16.7)	439 (13.6)	1.25 (1.10–1.43)		726 (16.8)	517 (13.3)		1.29 (1.15–1.45)	
	AA	4 (8.5)	1 (0.1)	2.01 (0.50–8.06)		21 (0.6)	11 (0.3)	2.09 (1.09–4.03)		25 (0.6)	12 (0.3)		1.99 (1.16–3.41)	
	per allele			1.43 (1.15–1.78)	3.0×10^−3^			1.27 (1.12–1.44)	2.0×10^−4^				1.30 (1.17–1.45)	3.1×10^−6^
rs184577 Chr2 (*C2orf58)*	GG	520 (61.9)	368 (56.9)	1.00		2104 (60.3)	1824 (56.4)	1.00		2624 (60.6)	2192 (56.5)	0.25	1.00	
	GA	278 (33.1)	234 (36.2)	0.86 (0.71–1.03)		1212 (34.7)	1231 (38.1)	0.83 (0.75–0.92)		1490 (34.4)	1465 (37.8)		0.83 (0.76–0.91)	
	AA	42 (5.0)	45 (7.0)	0.67 (0.46–0.96)		174 (5.0)	179 (5.5)	0.80 (0.64–0.99)		216 (5.0)	224 (5.8)		0.77 (0.64–0.93)	
	per allele			0.84 (0.73–0.97)	1.5×10^−2^			0.86 (0.79–0.93)	8.6×10^−5^				0.85 (0.79–0.91)	3.6×10^−6^

1 P-value was calculated based on the 1-degree of freedom score test.

SNPs in two additional regions had P-values<10^−5^ for breast cancer risk associations for *BRCA2* mutation carriers (Table 2). The magnitude of associations for both SNPs was similar in the discovery and second stage samples. In the combined analysis of all samples, the minor allele of rs619373, located in *FGF13* (Xq26.3), was associated with higher breast cancer risk (HR = 1.30, 95% CI 1.17–1.45, P = 3.1×10^−6^). The minor allele of rs184577, located in *CYP1B1-AS1* (2p22–p21), was associated with lower breast cancer risk (HR = 0.85, 95% CI 0.79–0.91, P = 3.6×10^−6^). These findings were consistent across countries (P for heterogeneity between country strata = 0.39 and P = 0.30, respectively; Figure S6). There was no evidence that the HR estimates for rs619373 and rs184577 change with age of the *BRCA2* mutation carriers (P for the genotype-age interaction = 0.80 and P = 0.40, respectively) and no evidence of survival bias for either SNP (rs619373: HR = 1.35, 95% CI 1.20–1.53, P = 1.5×10^−6^ and rs184577: HR = 0.86, 95% CI 0.79–0.93, P = 2.0×10^−4^, after excluding prevalent cases). The estimates for risk of ER-negative and ER-positive breast cancer were not significantly different (P for heterogeneity between tumor subtypes = 0.79 and 0.67, respectively). When associations were evaluated under a competing risks model, there was no evidence of association with ovarian cancer risk for SNPs rs9348512 at 6p24, rs619373 in *FGF13* or rs184577 at 2p22 and the breast cancer associations were virtually unchanged (Table S4).

Gene set enrichment analysis confirmed that strong associations exist for known breast cancer susceptibility loci and the novel loci identified here (gene-based P<1×10^−5^). The pathways most strongly associated with breast cancer risk that contained statistically significant SNPs included those related to ATP binding, organ morphogenesis, and several nucleotide bindings (pathway-based P<0.05).

To begin to determine the functional effect of rs9348512, we examined associations of expression levels of any nearby gene in breast tumors with the minor A allele. Using data from The Cancer Genome Atlas, we found that the A allele of rs9348512 was strongly associated with mRNA levels of *GCNT2* in breast tumors (*p* = 7.3×10^−5^).

**Figure 2:**
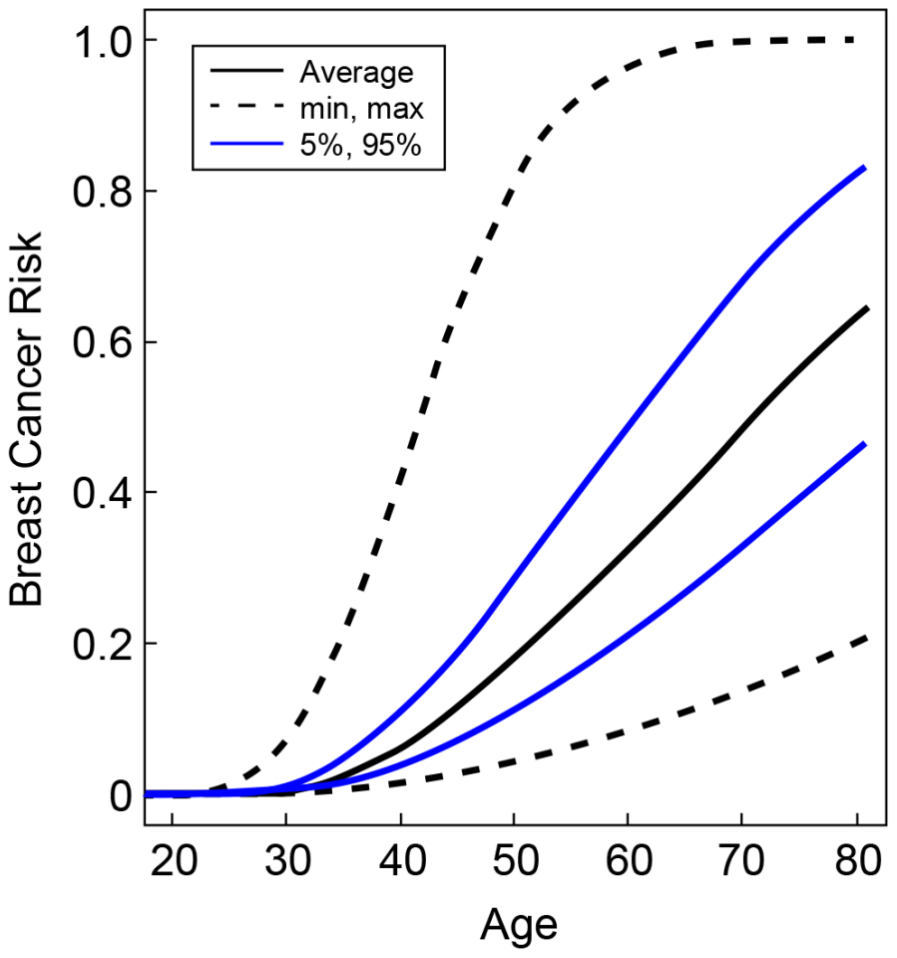
Predicted breast cancer risks for *BRCA2* mutation carriers by the combined SNP profile distributions. Based on the known breast cancer susceptibility loci at *FGFR2, TOX3*, 12p11, 5q11, *CDKN2A/B, LSP1*, 8q24, *ESR1*, *ZNF365*, 3p24, 12q24, 5p12, 11q13 and the newly identified *BRCA2* modifier locus at 6p24. The figure shows the risks at the 5^th^ and 95^th^ percentiles of the combined genotyped distribution as well as minimum, maximum and average risks.

The hazard ratios for the percentiles of the combined genotype distribution of loci associated with breast cancer risk in *BRCA2* mutation carriers were translated into absolute breast cancer risks under the assumption that SNPs interact multiplicatively. Based on our results for SNPs in *FGFR2, TOX3*, 12p11, 5q11, *CDKN2A/B, LSP1*, 8q24, *ESR1*, *ZNF365*, 3p24, 12q24, 5p12, 11q13 and also the 6p24 locus, the 5% of the *BRCA2* mutation carriers at lowest risk were predicted to have breast cancer risks by age 80 in the range of 21–47% compared to 83–100% for the 5% of mutation carriers at highest risk on the basis of the combined SNP profile distribution (Figure 2). The breast cancer risk by age 50 was predicted to be 4–11% for the 5% of the carriers at lowest risk compared to 29–81% for the 5% at highest risk.

## Discussion

In the largest assemblage of *BRCA2* mutation carriers, we identified a novel locus at 6q24 that is associated with breast cancer risk, and noted two potential SNPs of interest at Xq26 and 2p22. We also replicated associations with known breast cancer susceptibility SNPs previously reported in the general population and in *BRCA2* mutation carriers. For the 12p11 (*PTHLH*), 5q11 (*MAP3K1)*, and 9p21 (*CDKN2A/B*), we found uncorrelated SNPs that had stronger associations than the originally identified SNP in the breast cancer susceptibility region that should be replicated in the general population. In *BRCA2* mutation carriers, evidence for a breast cancer association with genetic variants in *PTHLH* has been restricted previously to ER-negative tumors [25]; however, the novel susceptibility variant we reported here was associated with risk of ER+ and ER- breast cancer.

The novel SNP rs9348512 (6p24) is located in a region with no known genes (Figure 1). *C6orf218*, a gene encoding a hypothetical protein LOC221718, and a possible tumor suppressor gene, *TFAP2A*, are within 100 kb of rs9348512. *TFAP2A* encodes the AP-2α transcription factor that is normally expressed in breast ductal epithelium nuclei, with progressive expression loss from normal, to ductal carcinoma *in situ*, to invasive cancer [26], [27]. AP-2α also acts as a tumor suppressor via negative regulation of MYC [28] and augmented p53-dependent transcription [29]. However, the minor allele of rs9348512 was not associated with gene expression changes of TFAP2A in breast cancer tissues in The Cancer Genome Atlas (TCGA) data; this analysis might not be informative since expression of TFAP2A in invasive breast tissue is low [26], [27]. Using the TCGA data and a 1 Mb window, expression changes with genotypes of rs9348512 were observed for *GCNT2*, the gene encoding the enzyme for the blood group I antigen glucosaminyl (N-acetyl) transferase 2. GCNT2, recently found to be overexpressed in highly metastatic breast cancer cell lines [30] and basal-like breast cancer [31], interacts with TGF-β to promote epithelial-to-mesenchymal transition, enhancing the metastatic potential of breast cancer [31]. An assessment of alterations in expression patterns in normal breast tissue from *BRCA2* mutation carriers by genotype are needed to further evaluate the functional implications of rs9348512 in the breast tumorigenesis of *BRCA2* mutation carriers.

To determine whether the breast cancer association with rs9348512 was limited to *BRCA2* mutation carriers, we compared results to those in the general population genotyped by BCAC and to *BRCA1* mutation carriers in CIMBA. No evidence of an associations between rs9348512 and breast cancer risk was observed in the general population (OR = 1.00, 95% CI 0.98–1.02, P = 0.74) [14], nor in *BRCA1* mutation carriers (HR = 0.99, 95% CI 0.94–1.04, P = 0.75) [13]. Stratifying cases by ER status, there was no association observed with ER-subtypes in either the general population or among *BRCA1* mutation carriers (BCAC: ER positive P = 0.89 and ER negative P = 0.60; CIMBA *BRCA1*: P = 0.49 and P = 0.99, respectively). For the two SNPs associated with breast cancer with P<10^−5^, neither rs619373, located in *FGF13* (Xq26.3), nor rs184577, located in *CYP1B1-AS1* (2p22-p21), was associated with breast cancer risk in the general population [14] or among *BRCA1* mutation carriers [13]. The narrow CIs for the overall associations in the general population and in *BRCA1* mutation carriers rule out associations of magnitude similar to those observed for *BRCA2* mutation carriers. The consistency of the association in the discovery and replication stages and by country, the strong quality control measures and filters, and the clear cluster plot for rs9348512 suggest that our results constitute the discovery of a novel breast cancer susceptibility locus specific to *BRCA2* mutation carriers rather than a false positive finding. Replicating this SNP in an even larger population of *BRCA2* mutation carriers would be ideal, but not currently possible because we know of no investigators with appropriate data and germline DNA from *BRCA2* mutation carriers who did not contribute their mutation carriers to iCOGS. However, CIMBA studies continue to recruit individuals into the consortium.

rs9348512 (6p24) is the first example of a common susceptibility variant identified through GWAS that modifies breast cancer risk specifically in *BRCA2* mutation carriers. Previously reported *BRCA2*-modifying alleles for breast cancer, including those in *FGFR2*, *TOX3*, *MAP3K1*, *LSP1*, 2q35, *SLC4A7*, 5p12, 1p11.2, *ZNF365*, and 19p13.1 (ER-negative only) [18], [32], [33], are also associated with breast cancer risk in the general population and/or *BRCA1* mutation carriers. Knowledge of the 6p24 locus might provide further insights into the biology of breast cancer development in *BRCA2* mutation carriers. Additional variants that are specific modifiers of breast cancer risk in *BRCA2* carriers may yet be discovered; their detection would require assembling larger samples of *BRCA2* mutation carriers in the future.

While individually each of the SNPs associated with breast cancer in *BRCA2* mutation carriers are unlikely to be used to guide breast cancer screening and risk-reducing management strategies, the combined effect of the general and *BRCA2*-specific breast cancer susceptibility SNPs might be used to tailor manage subsets of *BRCA2* mutation carriers. Taking into account all loci associated with breast cancer risk in *BRCA2* mutation carriers from the current analysis, including the 6p24 locus, the 5% of the *BRCA2* mutation carriers at lowest risk were predicted to have breast cancer risks by age 80 in the range of 21–47% compared to 83–100% for the 5% of mutation carriers at highest risk on the basis of the combined SNP profile distribution. These results might serve as a stimulus for prospective trials of the clinical utility of such modifier panels.

## Supporting Information

Figure S1 Cluster plots for SNPs (A.) rs9348512, (B.) rs619373, and (C.) rs184577.
(TIF)

Figure S2 Multidimensional scaling plots of the top two principal components of genomic ancestry of all eligible *BRCA2* iCOGS samples plotted with the HapMap CEU, ASI, and YRI samples: (A.) samples from Finland and *BRCA2* 6174delT carriers highlighted, and (B.) samples, indicated in red, with >19% non-European ancestry were excluded.
(TIF)

Figure S3 Quantile–quantile plot comparing expected and observed distributions of P-values. Results displayed (A) for the complete sample, (B) after excluding samples from the GWAS discovery stage, and (C) for the complete sample and a set of SNPs from the iCOGS array that were selected independent from the results of the *BRCA2* mutation carriers.
(TIF)

Figure S4 Manhattan plot of P-values by chromosomal position for 18,086 SNPs selected on the basis of a previously published genome-wide association study of *BRCA2* mutation carriers. Breast cancer associations results based on 4,330 breast cancer cases and 3,881 unaffected *BRCA2* carriers.
(TIF)

Figure S5 Forest plot of the country-specific, per-allele hazard ratios (HR) and 95% confidence intervals for the association between breast cancer and rs9348512 genotypes.
(TIF)

Figure S6 Forest plot of the country-specific, per-allele hazard ratios (HR) and 95% confidence intervals for the association with breast cancer for (A.) rs619373 and (B.) rs184577 genotypes.
(TIF)

Table S1 Quality control filtering steps for *BRCA2* mutation carriers and SNPs on the COGs array.
(DOC)

Table S2 Description of breast cancer affected and unaffected *BRCA2* carriers included in the final analysis of the COGs array SNPs.
(DOC)

Table S3 Breast cancer hazards ratios (HR) and 95% confidence intervals (CI) for all SNPs with P<10^−3^ in a 500 Mb region around rs9348512 on 6p24 among *BRCA2* mutation carriers.
(DOC)

Table S4 Associations with SNPs at 6p24, *FGF13* and 2p22 and breast and ovarian cancer risk using a competing risk analysis model.
(DOC)
