# Supplementary figures and images for: Identification of a BRCA2-Specific Modifier Locus at 6p24 Related to Breast Cancer Risk
Source: PLoS Genet. 2013 Mar 27;9(3):e1003173. doi: 10.1371/journal.pgen.1003173 (PMC3609647; doi:10.1371/journal.pgen.1003173)

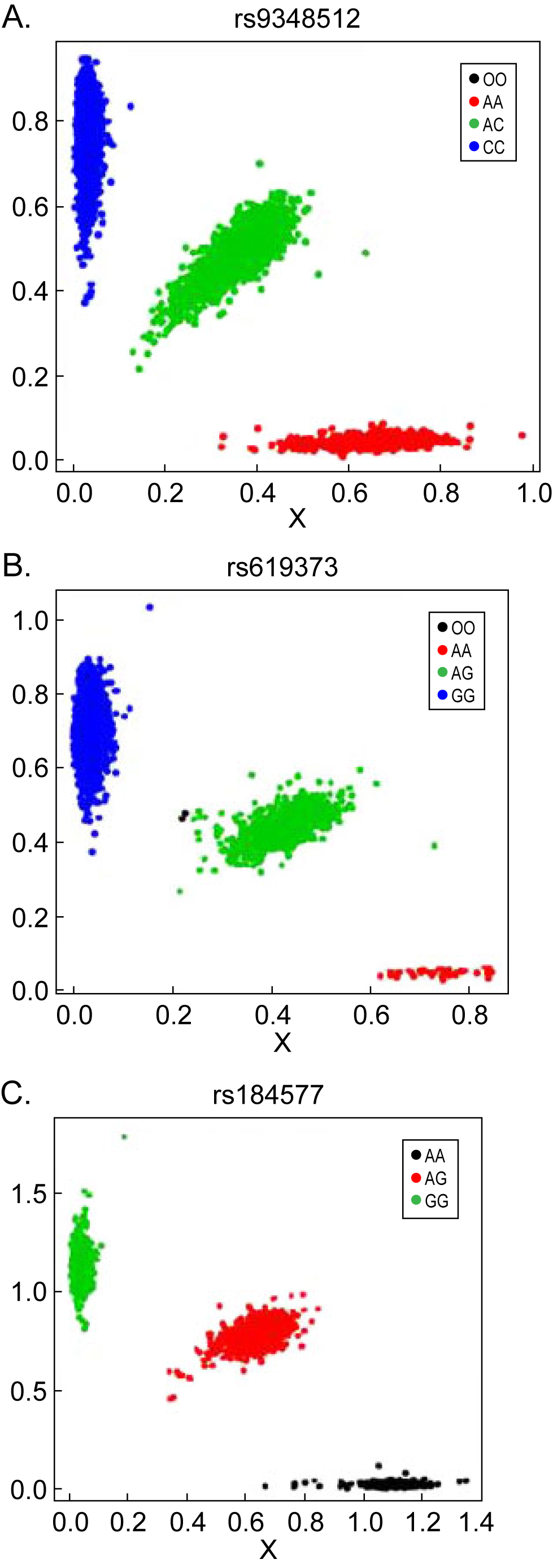

Supplement: Figure S1 — Cluster plots for SNPs (A.) rs9348512, (B.) rs619373, and (C.) rs184577. (TIF) [file pgen.1003173.s001.tif]

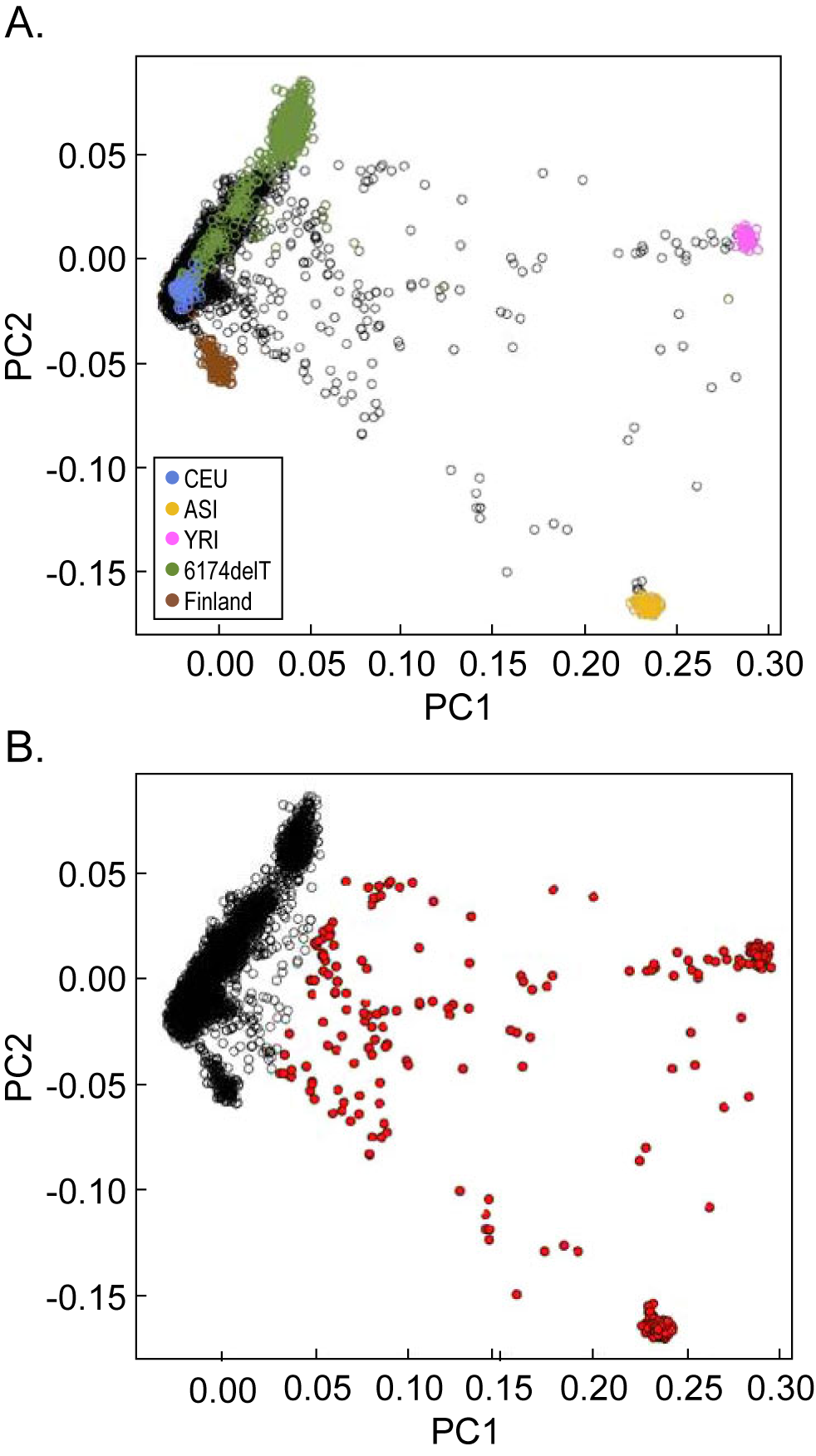

Supplement: Figure S2 — Multidimensional scaling plots of the top two principal components of genomic ancestry of all eligible BRCA2 iCOGS samples plotted with the HapMap CEU, ASI, and YRI samples: (A.) samples from Finland and BRCA2 6174delT carriers highlighted, and (B.) samples, indicated in red, with >19% non-European ancestry were excluded. (TIF) [file pgen.1003173.s002.tif]

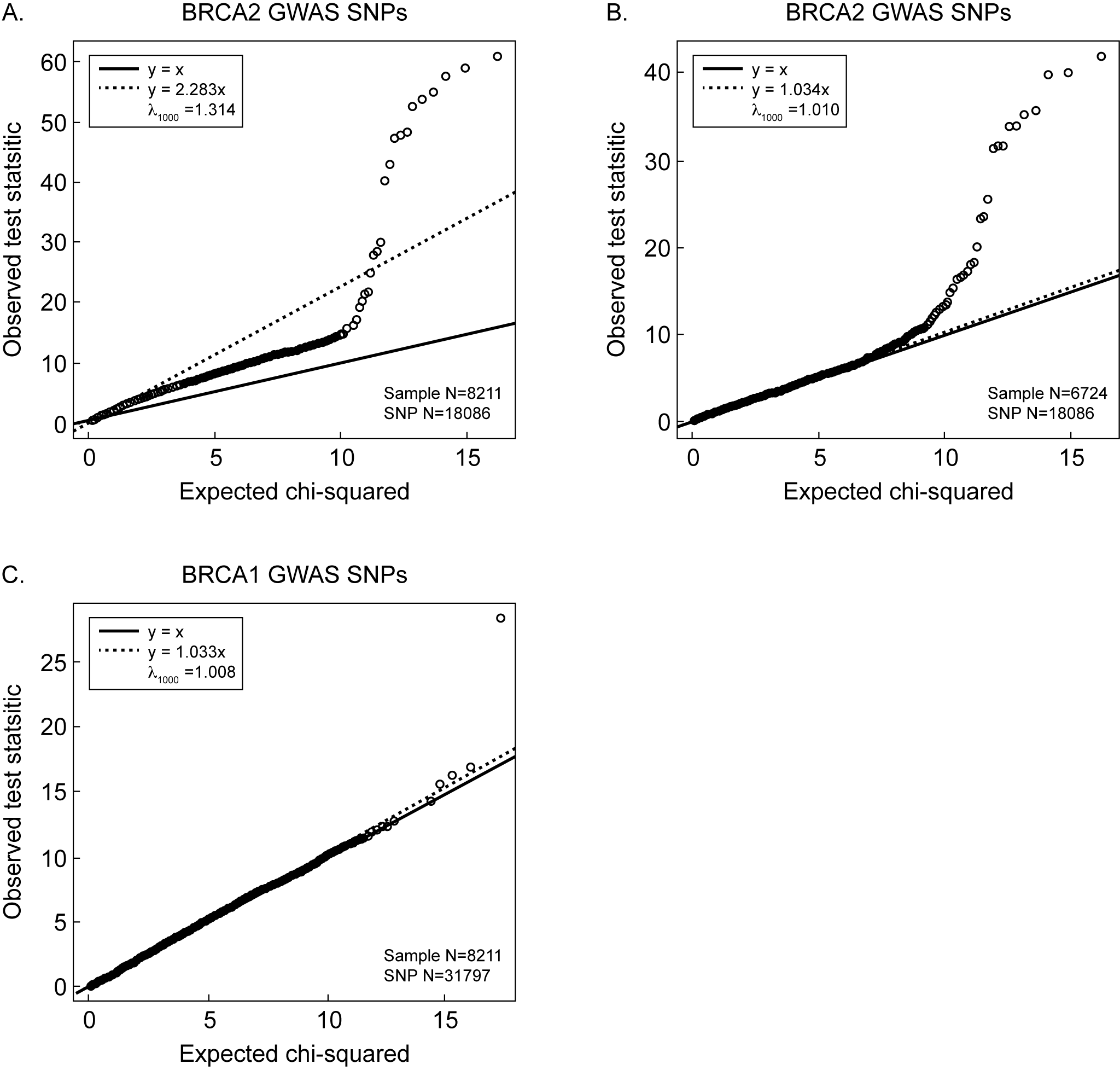

Supplement: Figure S3 — Quantile–quantile plot comparing expected and observed distributions of P-values. Results displayed (A) for the complete sample, (B) after excluding samples from the GWAS discovery stage, and (C) for the complete sample and a set of SNPs from the iCOGS array that were selected independent from the results of the BRCA2 mutation carriers. (TIF) [file pgen.1003173.s003.tif]

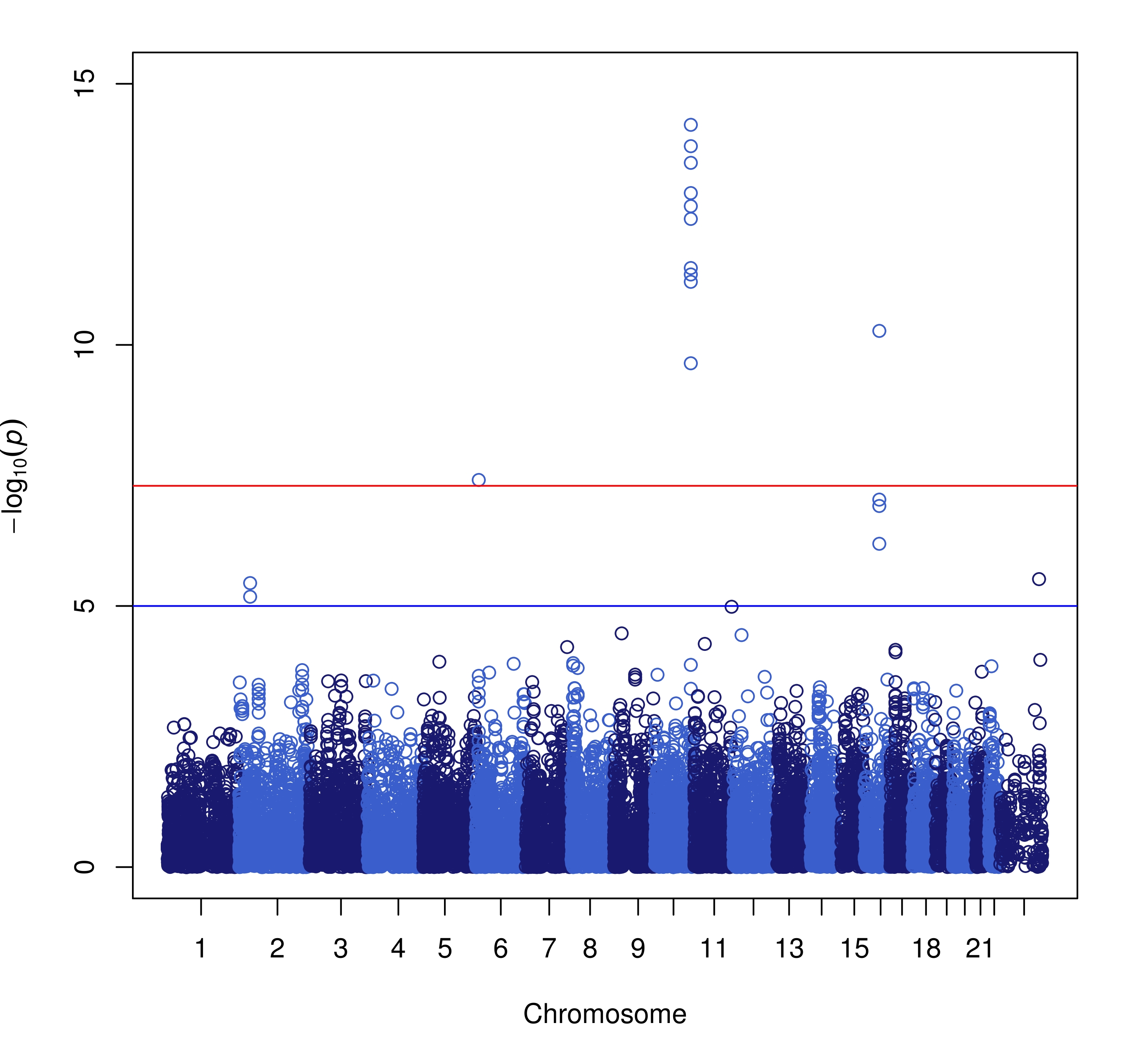

Supplement: Figure S4 — Manhattan plot of P-values by chromosomal position for 18,086 SNPs selected on the basis of a previously published genome-wide association study of BRCA2 mutation carriers. Breast cancer associations results based on 4,330 breast cancer cases and 3,881 unaffected BRCA2 carriers. (TIF) [file pgen.1003173.s004.tif]

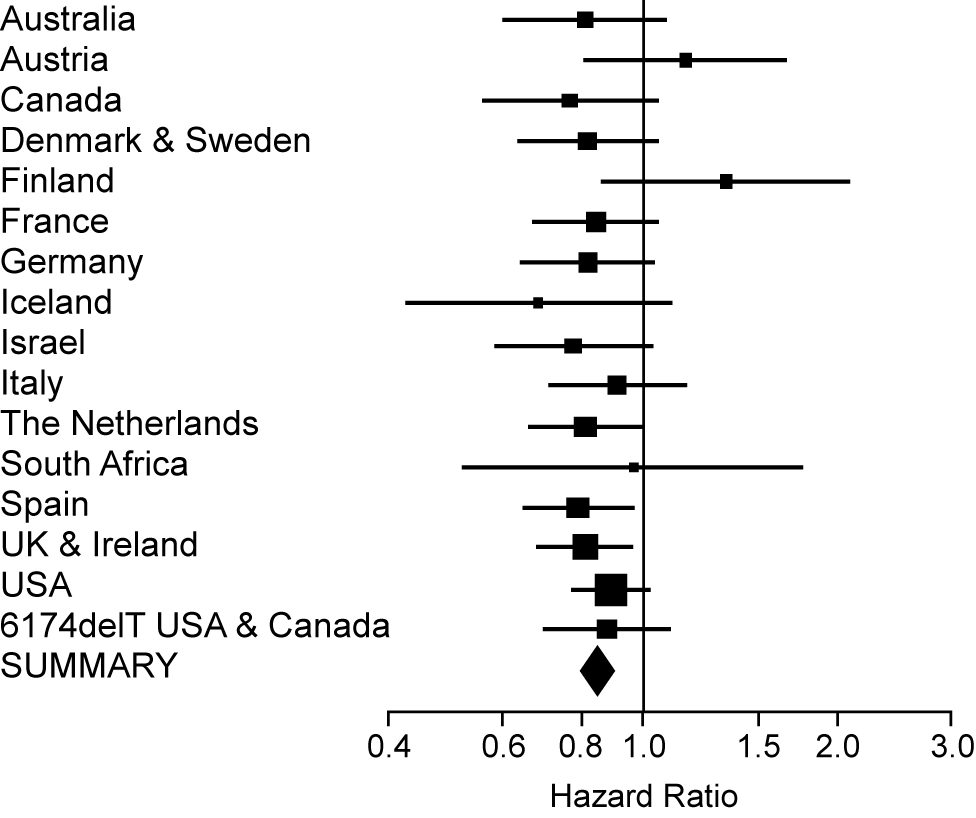

Supplement: Figure S5 — Forest plot of the country-specific, per-allele hazard ratios (HR) and 95% confidence intervals for the association between breast cancer and rs9348512 genotypes. (TIF) [file pgen.1003173.s005.tif]

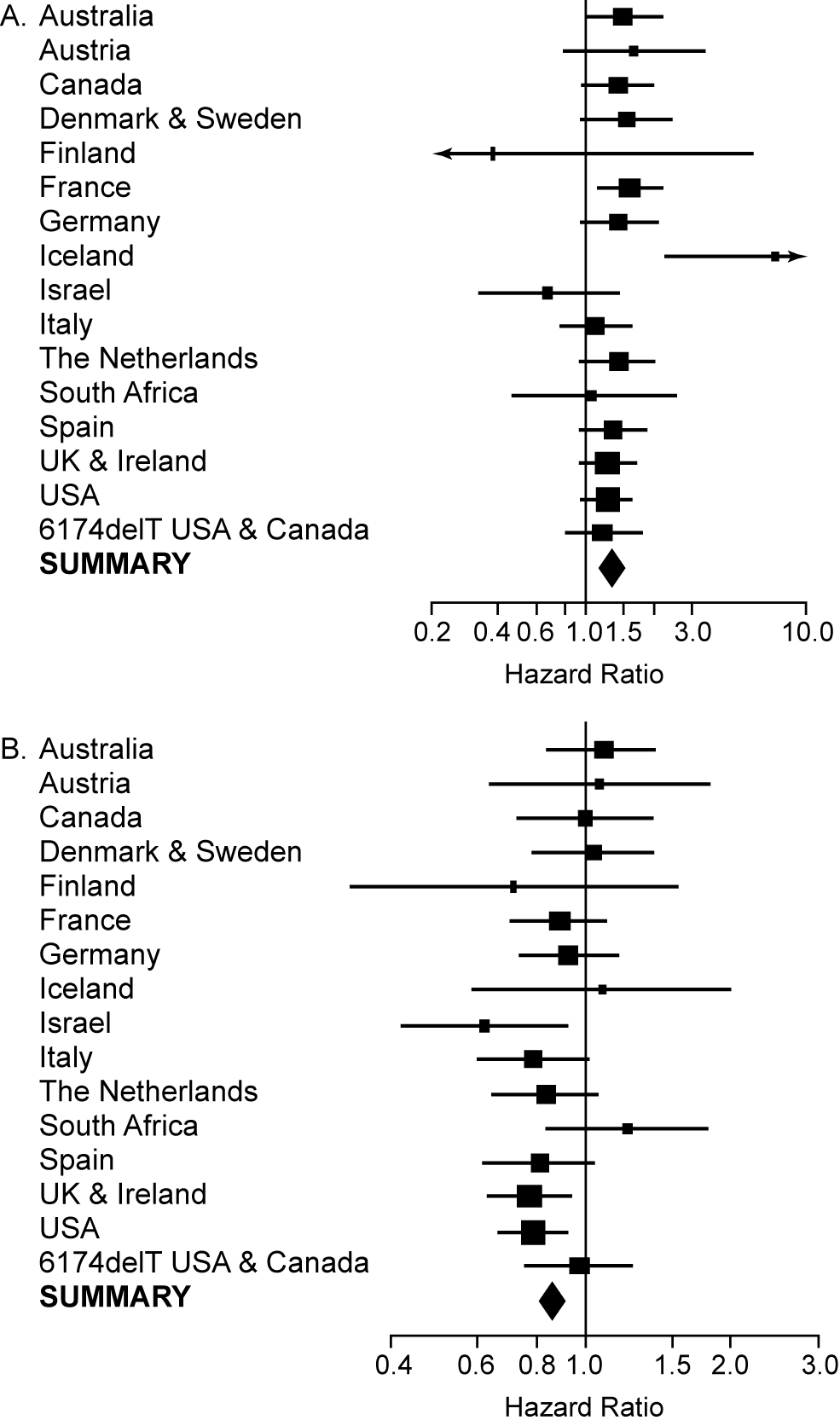

Supplement: Figure S6 — Forest plot of the country-specific, per-allele hazard ratios (HR) and 95% confidence intervals for the association with breast cancer for (A.) rs619373 and (B.) rs184577 genotypes. (TIF) [file pgen.1003173.s006.tif]
