# Supplementary material for: Identification of a BRCA2-Specific Modifier Locus at 6p24 Related to Breast Cancer Risk
Source: PLoS Genet. 2013 Mar 27;9(3):e1003173. doi: 10.1371/journal.pgen.1003173 (PMC3609647; doi:10.1371/journal.pgen.1003173)
Supplement: Table S1 — Quality control filtering steps for BRCA2 mutation carriers and SNPs on the COGs array. (DOC) [file pgen.1003173.s007.doc]

**Table S1**. Description of breast cancer affected and unaffected *BRCA2* carriers included in the final analysis of the COGs array SNPs

| **Factor** | **Affected (n=4,330)** | | **Unaffected (n=3,881)** | |
| --- | --- | --- | --- | --- |
|  | **N** | **%** | **N** | **%** |
| **Age at Censoring** |  |  |  |  |
| <40 | 1,545 | 35.7 | 1,607 | 41.4 |
| 40-49 | 1,651 | 38.1 | 1,025 | 26.4 |
| 50-59 | 799 | 18.5 | 712 | 18.4 |
| 60+ | 335 | 7.7 | 537 | 13.8 |
| **Ashkenazi Jewish Ancestry** | | | | |
| No | 3,988 | 92.1 | 3,433 | 88.5 |
| Yes | 342 | 7.9 | 448 | 11.5 |
| **BRCA2*6174delT Mutation Carrier** | | | | |
| Yes | 435 | 10.0 | 584 | 15.0 |
| No | 3,895 | 90.0 | 3,297 | 85.0 |
| **Country of Residence** |  |  |  |  |
| Australia | 288 | 6.7 | 200 | 5.2 |
| Austria | 123 | 2.8 | 77 | 2.0 |
| Canada | 153 | 3.5 | 150 | 3.9 |
| Denmark & Sweden | 158 | 3.6 | 198 | 5.1 |
| Finland | 66 | 1.5 | 55 | 1.4 |
| France | 491 | 11.3 | 209 | 5.4 |
| Germany | 365 | 8.4 | 198 | 5.1 |
| Iceland | 102 | 2.4 | 25 | 0.6 |
| Israel | 108 | 2.5 | 166 | 4.3 |
| Italy | 353 | 8.2 | 174 | 4.5 |
| South Africa | 93 | 2.1 | 53 | 1.4 |
| Spain | 328 | 7.6 | 293 | 7.5 |
| The Netherlands | 260 | 6.0 | 492 | 12.7 |
| United Kingdom & Ireland | 483 | 11.2 | 560 | 14.4 |
| USA | 959 | 22.1 | 1,031 | 26.6 |
| **Study** |  |  |  |  |
| BCFR | 197 | 4.5 | 152 | 3.9 |
| BIDMC | 4 | 0.09 | 5 | 0.1 |
| BMBSA | 93 | 2.1 | 53 | 1.4 |
| BRICOH | 48 | 1.1 | 80 | 2.1 |
| CBCS | 46 | 1.1 | 49 | 1.3 |
| CNIO | 113 | 2.6 | 113 | 2.9 |
| COH | 65 | 1.5 | 42 | 1.1 |
| CONSIT TEAM | 263 | 6.1 | 137 | 3.5 |
| DFCI | 55 | 1.3 | 79 | 2.0 |
| DKFZ | 14 | 0.3 | 11 | 0.3 |
| EMBRACE | 478 | 11.0 | 547 | 14.1 |
| FCCC | 19 | 0.4 | 35 | 0.9 |
| GC-HBOC | 351 | 8.1 | 186 | 4.8 |
| GEMO | 523 | 12.1 | 226 | 5.8 |
| GOG | 152 | 3.5 | 161 | 4.1 |
| HCSC | 59 | 1.4 | 54 | 1.4 |
| HEBON | 260 | 6.0 | 492 | 12.7 |
| HEBCS | 66 | 1.5 | 55 | 1.4 |
| HVH | 34 | 0.8 | 25 | 0.6 |
| ICO | 122 | 2.8 | 102 | 2.6 |
| ILUH | 103 | 2.4 | 26 | 0.7 |
| INHERIT | 26 | 0.6 | 23 | 0.6 |
| IOVHBOCS | 90 | 2.1 | 37 | 1.0 |
| kConFab | 254 | 5.9 | 182 | 4.7 |
| MAGIC | 8 | 0.2 | 22 | 0.6 |
| MAYO | 80 | 1.8 | 61 | 1.6 |
| MCGILL | 12 | 0.3 | 15 | 0.4 |
| MSKCC | 121 | 2.8 | 97 | 2.5 |
| MUV | 123 | 2.8 | 77 | 2.0 |
| NCI | 22 | 0.5 | 61 | 1.6 |
| NICCC | 61 | 1.4 | 108 | 2.8 |
| OCGN | 65 | 1.5 | 112 | 2.9 |
| OSU CCG | 33 | 0.8 | 28 | 0.7 |
| OUH | 89 | 2.1 | 117 | 3.0 |
| SMC | 47 | 1.1 | 57 | 1.5 |
| SWE-BRCA | 23 | 0.5 | 31 | 0.8 |
| UCHICAGO | 25 | 0.6 | 12 | 0.3 |
| UCLA | 15 | 0.3 | 26 | 0.7 |
| UCSF | 16 | 0.4 | 11 | 0.3 |
| UKGRFOCR | 4 | 0.09 | 13 | 0.3 |
| UPENN | 134 | 3.1 | 105 | 2.7 |
| WCP | 17 | 0.4 | 54 | 1.4 |
