# Supplementary material for: Identification of a BRCA2-Specific Modifier Locus at 6p24 Related to Breast Cancer Risk
Source: PLoS Genet. 2013 Mar 27;9(3):e1003173. doi: 10.1371/journal.pgen.1003173 (PMC3609647; doi:10.1371/journal.pgen.1003173)
Supplement: Table S2 — Description of breast cancer affected and unaffected BRCA2 carriers included in the final analysis of the COGs array SNPs. (DOC) [file pgen.1003173.s008.doc]

**Table S2**. Quality control filtering steps for *BRCA2* mutation carriers and SNPs on the COGs array

| **Sample Data Cleaning Steps/Exclusion Reasons** | **No. of samples** | **Remaining No. of Samples** | **Data Cleaning Steps for SNPs** | **No. of SNPs** | **Remaining No. of SNPs** |
| --- | --- | --- | --- | --- | --- |
| **Total Eligible Samples on Manifest with Genotype Data** |  | **10,048** | **Total SNPs on COGs Array** |  | **211,155** |
| Ineligible based on phenotypic data | 211 | 9,837 | Y chromosome SNPs | 79 | 211,076 |
| Self-reported non-CEU ethnicity | 531 | 9,306 | Call rate <95% | 4,446 | 206,630 |
| Incorrect gender based on genotype | 34 | 9,272 | HWE(stratified) P-value <10^-7^ | 1,845 | 204,785 |
| Call rate <95% | Heterozygosity: P-value <10^-6^ | 300 | 8,972 | Monomorphic markers | 3,853 | 200,932 |
| >19% inferred non-CEU ancestry | 166 | 8,806 | Unreliable SNP genotypes | 1 | 200,931 |
| Discordant with previous CIMBA genotyping | 53 | 8,753 | SNPs with high discordance rate among known duplicates (list obtained from all members of COGs consortia) | 23 | 200,908 |
| Consistent duplicate pairs (one sample excluded | 498 | 8,255 |
| Inconsistent duplicate pairs (both samples excluded) | 44 | 8,211 |
| **Totals After Filtering** | 1,837 | **8,211** |  | 10,439 | **200,908** |
