# Supplementary material for: Identification of a BRCA2-Specific Modifier Locus at 6p24 Related to Breast Cancer Risk
Source: PLoS Genet. 2013 Mar 27;9(3):e1003173. doi: 10.1371/journal.pgen.1003173 (PMC3609647; doi:10.1371/journal.pgen.1003173)
Supplement: Table S3 — Breast cancer hazards ratios (HR) and 95% confidence intervals (CI) for all SNPs with P<10−3 in a 500 Mb region around rs9348512 on 6p24 among BRCA2 mutation carriers. (DOC) [file pgen.1003173.s009.doc]

**Table S3.** Breast cancer hazards ratios (HR) and 95% confidence intervals (CI) for all SNPs with P<10-3 in a 500Mb region around rs9348512 on 6p24 among *BRCA2* mutation carriers

| **SNP** | **Position** | **Type^1^** | **Major Allele** | **Minor Allele** | **r^2^ with rs9348512** | **Minor Allele Freq.** | **r^2^ imputation** | **P-value^2^** |
| --- | --- | --- | --- | --- | --- | --- | --- | --- |
| rs9348512 | 10564692 | typed | C | A | 1.00 | 0.34 | 1.00 | 4.4x10^-8^ |
| rs9358529 | 10563215 | imputed | A | C | 0.86 | 0.31 | 0.96 | 8.2x10^-7^ |
| rs303067 | 10548212 | imputed | A | T | 0.71 | 0.40 | 0.95 | 1.9x10^-6^ |
| rs1348 | 10557244 | imputed | T | C | 0.51 | 0.21 | 0.96 | 1.0x10^-5^ |
| rs9366443 | 10565096 | imputed | C | T | 0.72 | 0.41 | 0.94 | 3.7x10^-5^ |
| rs9460713 | 10555412 | imputed | C | T | 0.49 | 0.20 | 0.96 | 4.9x10^-5^ |
| rs9466289 | 10555931 | imputed | T | C | 0.50 | 0.20 | 0.97 | 5.4x10^-5^ |
| 6-10546956 | 10546956 | imputed | A | AGG | 0.48 | 0.41 | 0.84 | 5.9x10^-5^ |
| rs9466290 | 10555941 | imputed | G | A | 0.49 | 0.20 | 0.96 | 6.4x10^-5^ |
| rs3911709 | 10559876 | imputed | G | A | 0.45 | 0.26 | 0.96 | 7.3x10^-5^ |
| 6-10557995 | 10557995 | imputed | GTAT | G | 0.49 | 0.20 | 0.95 | 7.4x10^-5^ |
| rs9295542 | 10565669 | imputed | A | G | 0.61 | 0.46 | 0.91 | 8.2x10^-5^ |
| rs6908107 | 10559449 | imputed | C | G | 0.61 | 0.46 | 0.98 | 8.8x10^-5^ |
| rs35076407 | 10563463 | imputed | T | C | 0.62 | 0.45 | 0.98 | 9.2x10^-5^ |
| rs602199 | 10554912 | imputed | C | G | 0.60 | 0.39 | 0.94 | 9.3x10^-5^ |
| rs7738545 | 10563318 | imputed | C | T | 0.62 | 0.45 | 0.99 | 9.7x10^-5^ |
| rs303074 | 10560093 | imputed | G | A | 0.61 | 0.46 | 0.98 | 1.0x10^-4^ |
| rs78113724 | 10543366 | imputed | G | A | 0.45 | 0.24 | 0.97 | 1.1x10^-4^ |
| rs303073 | 10560449 | imputed | A | G | 0.61 | 0.46 | 0.98 | 1.3x10^-4^ |
| rs4712668 | 10562060 | typed | G | T | 0.61 | 0.45 | 1.00 | 1.4x10^-4^ |
| rs75769093 | 10551514 | imputed | C | A | 0.54 | 0.47 | 0.90 | 1.5x10^-4^ |
| rs303070 | 10551187 | imputed | G | T | 0.56 | 0.48 | 0.92 | 1.9x10^-4^ |
| rs9393239 | 10550632 | imputed | C | T | 0.43 | 0.21 | 0.98 | 2.0x10^-4^ |
| rs303061 | 10538157 | typed | T | C | 0.41 | 0.24 | 1.00 | 2.1x10^-4^ |
| rs4097280 | 10561081 | imputed | G | A | 0.58 | 0.44 | 0.95 | 2.3x10^-4^ |
| rs4710998 | 10541811 | typed | A | G | 0.39 | 0.22 | 1.00 | 2.8x10^-4^ |
| rs6907578 | 10532198 | imputed | T | A | 0.53 | 0.43 | 0.86 | 3.7x10^-4^ |
| 6-10550552 | 10550552 | imputed | G | T | 0.55 | 0.47 | 0.90 | 3.9x10^-4^ |
| rs56365413 | 10540162 | imputed | C | T | 0.40 | 0.20 | 0.98 | 4.0x10^-4^ |
| 6-10545251 | 10545251 | imputed | GTTGTTGTT | G | 0.38 | 0.21 | 0.99 | 4.4x10^-4^ |
| rs303068 | 10549297 | imputed | A | C | 0.56 | 0.48 | 0.92 | 4.5x10^-4^ |
| rs6923826 | 10532295 | imputed | C | G | 0.50 | 0.42 | 0.86 | 4.8x10^-4^ |
| rs12175352 | 10545445 | imputed | T | C | 0.38 | 0.21 | 0.99 | 4.8x10^-4^ |
| rs303065 | 10541072 | imputed | C | T | 0.39 | 0.20 | 0.99 | 5.0x10^-4^ |
| rs303064 | 10540524 | typed | C | T | 0.38 | 0.20 | 1.00 | 5.2x10^-4^ |
| rs9295535 | 10547954 | typed | T | C | 0.40 | 0.21 | 1.00 | 6.2x10^-4^ |
| rs115262601 | 10526048 | imputed | A | C | 0.01 | 0.01 | 0.44 | 6.6x10^-4^ |
| rs6924202 | 10532454 | imputed | C | T | 0.56 | 0.39 | 0.80 | 6.7x10^-4^ |
| rs12526269 | 10536199 | imputed | T | A | 0.37 | 0.21 | 0.98 | 7.1x10^-4^ |
| 6-10546510 | 10546510 | imputed | GC | G | 0.48 | 0.44 | 0.94 | 7.9x10^-4^ |
| rs303063 | 10538964 | imputed | C | T | 0.38 | 0.20 | 0.99 | 8.7x10^-4^ |

^1^Type indicates whether the SNP was genotyped or imputed.

^2^p-value was calculated based on the 1-degree of freedom score test
