# Supplementary material for: Identification of a BRCA2-Specific Modifier Locus at 6p24 Related to Breast Cancer Risk
Source: PLoS Genet. 2013 Mar 27;9(3):e1003173. doi: 10.1371/journal.pgen.1003173 (PMC3609647; doi:10.1371/journal.pgen.1003173)
Supplement: Table S4 — Associations with SNPs at 6p24, FGF13 and 2p22 and breast and ovarian cancer risk using a competing risk analysis model. (DOC) [file pgen.1003173.s010.doc]

**Table S4.** Associations with SNPs at 6p24, *FGF13* and 2p22 and breast and ovarian cancer risk using a competing risk analysis model

| **SNP rs No. Chr.** | **No. unaf-fected** | **No. breast cancer** | **No. ovarian cancer** | **Ovarian cancer** | | **Breast cancer** | |
| --- | --- | --- | --- | --- | --- | --- | --- |
|  |  |  |  | **HR (95%CI)** | **p-value** | **HR (95%CI)** | **p-value** |
| rs9348512  6p24 | 3432 | 4310 | 468 | 0.98 (0.84,1.13) | 0.74 | 0.84 (0.79,0.90) | 8.7x10^-8^ |
| rs619373  Xq26 | 3432 | 4307 | 468 | 0.98 (0.73,1.30) | 0.88 | 1.29 (1.15,1.44) | 7.1x10^-6^ |
| rs184577  2p22 | 3432 | 4311 | 468 | 1.01 (0.86,1.19) | 0.89 | 0.85 (0.79,0.92) | 1.4x10^-5^ |
